# Characterization and Rheological Properties of Ultra-High Molecular Weight Polyethylenes

**DOI:** 10.3390/polym16243501

**Published:** 2024-12-16

**Authors:** Alexander Ya. Malkin, Tatyana A. Ladygina, Sergey S. Gusarov, Dmitry V. Dudka, Anton V. Mityukov

**Affiliations:** 1 A.V. Topchiev Institute of Petrochemical Synthesis, Russian Academy of Sciences, 29, Leninskiy Prospect, 119991 Moscow, Russia; dudim2009@gmail.com (D.V.D.);; 2 N.N. Semenov Federal Research Center for Chemical Physics, Russian Academy of Sciences, 4, ul. Kosygina, 119991 Moscow, Russiaserezhagusarow@yandex.ru (S.S.G.)

**Keywords:** UHMWPE, GPC, MW, rheology, plasticity

## Abstract

The molecular characteristics and rheological properties of three UHMWPE samples were investigated. The high-temperature GPC method was used for characterizing UHMWPE samples used. The interpretation of the measurement results was based on calibration using the PS standard and the approximation of the PS data by linear and cubic polynomials, as well as on the data for linear PE. The assessment of the average MW and MWD depends on the choice of calibration method, so that different methods give different results. Only the results obtained using PS with cubic approximation are close to the characteristics offered by the manufacturer. It was also shown that the obtained MW characteristics depend on the dissolution time. The reason for this may be the presence of any processing-aid compounds or destruction of macromolecules. Measurements of the rheological properties were performed in creep modes for a wide range of shear stresses and harmonic oscillations. It was shown that even at 210 °C, UHMWPE does not flow, and the observed irreversible deformations are due to the plasticity of the polymer, i.e., UHMWPE is in an elastic–plastic state. The ultimate plastic deformations drop sharply with increasing MW of the polymer. The plasticity modulus for the highest molecular weight UHMWPE samples does not depend on stress. Measurements of viscoelastic characteristics confirmed that the terminal region of viscous flow cannot be reached under any conditions. Increasing the duration of holding the polymer at high temperature leads not to flow, but to the destruction of macromolecules.

## 1. Introduction

PE is one of the oldest and largest commercial products of the polymer industry. Several milestones can be identified in the development of PE. First, a method for high-pressure radical polymerization to produce low-density PE was developed in the 1930s. Then came the discovery of Ziegler–Natta catalysts and the production of high-density linear PE in the 1950s, intermediate versions of medium-pressure PE production, and the discovery of homogeneous metallocene catalysts in the 1990s to produce low-density linear PE. Finally, the production and introduction of ultra-high-molecular weight PE (UHMWPE) were put into technological practice. This class of materials traditionally includes linear PE with an MW of several (usually 3–6) million Da, i.e., degree of polymerization above 50 thousand. This is a material with high values of technical characteristics, a low coefficient of friction, capable of forming fibers, which are usually characterized by high values of the elastic modulus and extremely high strength, as well as a number of other outstanding properties.

UHMWPE belongs to the class of thermoplastic polymers. However, its application is limited due to the fact that even above the melting point (which is about 140 °C), its viscosity is extremely high and the melt is actually in a rubbery state. Therefore, it cannot be processed into products using traditional thermoplastic methods such as extrusion and injection molding. Many dozens of publications are devoted to the study of the structure, properties, and processing technology of UHMWPE, the results of which are summarized in reviews and encyclopedic publications (for example [1,2]). One of the main areas of application of UHMWPE is medicine [3], especially orthopedics [4,5]. The production of high-strength UHMWPE fibers (e.g., using gel technology [6]) has led to the creation of composites using these fibers for a variety of applications [7]. UHMWPE has been known for over 50 years. However, it remains one of the most mysterious polymers, continuing to attract the attention of researchers and technologists. New publications devoted to the study of UHMWPE are constantly appearing. This is facilitated by two objective problems. Firstly, it is not easy to characterize and thereby standardize the main property of UHMWPE—its molecular weight (MW). Secondly, it is necessary to improve the processability of the polymer, which requires a decrease in its viscosity. Both of these problems are related to the compatibility of UHMWPE with other substances, and the best in this regard are other PEs with traditional MW values of the order of tens of thousands of Da. The latter is achieved either by mechanical mixing or by technological methods of joint polymerization in a single volume [8] or mixing with low-viscosity components [9]. The present research continues this line of research using three different industrial grades of UHMWPE as an example. The specific task is to reliably determine the MWs of UHMWPE samples and evaluate their rheological properties.

## 2. Materials and Methods

The experiments were carried out with three UHMWPE samples: GUR-4120 (Celanese, Narrows, NC, USA), GUR-4150 (Celanese, USA), and UTEC 6540 (Braskem, Vamacari, Bahia, Brazil). According to the passport data of the manufacturers, the average MW values of these samples are 4 · 10^6^, 9 · 10^6^, and 8 · 10^6^ Da. To prevent thermal oxidation during their MW and MWD determination, 0.1% of the antioxidant Irganox 1010 was added. The molecular weight characteristics of the UHMWPE samples were measured by gel permeation chromatography (GPC) at 160 °C on a Tosoh HLC-8321 GPC/HT high-temperature chromatograph (Japan) equipped with a TSKgel GMHHR-H (20) HT2, 7.8 × 300 mm column. To record the signal during the molecular weight determination, a refractometer (RI detector) was used. Calibration curves were made by means of PS standards with narrow MWDs in the range from 2.7 · 10^3^ to 11.6 · 10^6^ but with different approximations. 1,2,4-Trichlorobenzene was used as a solvent. The sample weight was 1.0 mg/mL. Before the study, the samples were pre-dissolved in 1,2,4-trichlorobenzene at 160 °C with continuous stirring for 2.5–5.0 h. The elution rate was 1.0 mL/min.

Rheological properties were measured using a Thermo Scientific HAAKE RheoStress 600 rheometer (ThermoHaake, Dreieich, Germany) with a plane-to-plane measuring unit with a diameter of 20 mm (rough surfaces, distance between surfaces 1 mm). The experiments consisted of two types of measurements: determination of viscoelastic properties of samples in the mode of linear low-amplitude oscillations and creep measurements at specified stresses also in the linear region of viscoelastic behavior of the original UHMWPE samples. These tests were carried out at a temperature of 210 °C and in the stress range of 50–350 Pa, which was maintained for 3 h; elastic recovery usually was not measured, since despite the presence of an antioxidant in the sample, the material was destroyed during the holding time.

## 3. Results and Discussion

### 3.1. Determination of Molecular Characteristics of UHMWPE Samples

A review of the results of measurements of the intrinsic viscosity for branched PE was presented in publication [10] in the form of the Mark–Houwink equation for a set of different solvents, as well as for solutions of linear PE in decahydronaphthalene at 135 °C in the form of the following relationships:

For fractions by number-average MW:

(1) $$\left[\eta \right]=2.3\cdot 10^{-4}M_{w}^{0.82}$$

For fractions by weight-average MW:

(2) $$\left[\eta \right]=3.9\cdot 10^{-4}M_{w}^{0.74}$$

For unfractionated (whole) samples by average MW:

(3) $$\left[\eta \right]=2.55\cdot 10^{-4}M_{w}^{0.74}$$

For linear PEs with MWs on the order of tens of thousands of Da, the following Mark–Houwink equation is valid for solutions in decalin at 135 °C [11]:

(4) $$\left[\eta \right]=6.2\cdot 10^{-4}M_{w}^{0.70}$$

For solutions in diphenyl ether at 163.9 °C (theta solvent):

(5) $${\left[\eta \right]}_{\theta }=26.1\cdot 10^{-4}M_{w}^{0.5}$$

The difficulties of reliably determining the MWs and MWDs of UHMWPEs have led to various attempts to solve this problem by indirect methods. Thus, an indirect but fundamentally important method for determining the MWD is to calculate the latter using rheological data. In the general formulation, this is an ill-posed inverse problem of solving the integral equation linking the MWD with the rheological parameters [12]. However, with an a priori assignment of the function describing the MWD, such a calculation becomes possible. With this approach, the equation for the relaxation modulus, G_r_(t), is usually considered, which has the following form [13]:

(6) $$G_{r}\left(t\right)=G_{0}{\left\{\int_{\log M_{c}}^{\infty }F_{M}{\left(t,M\right)}^{1/\beta }wMd\log(M)\right\}}^{\beta }$$

where G_0_ is the elastic modulus in the rubbery plateau region, t is time, M_c_ is the MM corresponding to the formation of entanglements, F_M_(t, M) is the relaxation modulus for the monodisperse fraction with MW equal to M, which is expressed by the sum of the exponentials, and w is the weight fraction of the polymer. This approach is based on the double reptation model, for which the parameter β = 2.

The application of this approach was slightly modified for use in calculating the MWD of UHMWPE [14,15] and adapted to the relaxation of concentrated UHMWPE solutions in ethylene oligomers (paraffins). A review of possible methods for characterizing UHMWPE was summarized in the IUPAC report [16].

Below, the results of direct measurement of MW and MWD using a high-temperature gel chromatograph are described. In this case, these parameters were determined using different methods, using three different calibration procedures.

In the first calibration, linear approximation was used to calculate the MW characteristics. To recalculate the obtained MW characteristics from PS to PE, the coefficients K and α for PS and PE in 1,2,4-trichlorobenzene at 160 °C were taken according to the following formulas, respectively [16]:

(7) $$\left[\eta \right]=3.6\cdot 10^{-4}M_{w}^{0.655}$$

(8) $$\left[\eta \right]=2.1\cdot 10^{-4}M_{w}^{0.73}$$

**Figure 1:**
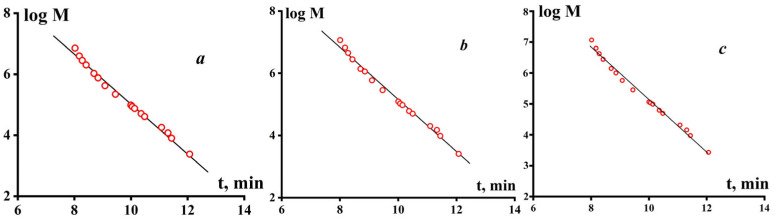
Calibration curves using PS standards and linear approximation with recalculating for PE (**a**), without recalculating (**b**), and cubic approximation without recalculating (**c**).

The second option for calculation in calibration of MW characteristics was made without taking into account the coefficients K and α for PS and PE in 1,2,4-trichlorobenzene at 160 °C. Therefore, the calibration with linear approximation made by means of PS standards was used. Both calibration curves are presented in Figure 1.

Experimental data can also be represented using a cubic approximation, expressed by the following equation:

(9) $$M=At^{3}+Bt^{2}+Ct+D$$

where A, B, C, and D are empirical constants.

For the third option of calibration, the coefficients K and α for PS and PE in 1,2,4-trichlorobenzene at 160 °C were not used.

When discussing the results, the first calibration will be referred to as “relative to PE”, the second calibration as “relative to PS–linear”, and the third calibration as “relative to PS–cubic”.

In addition to GPC, the viscometry method was used for UHMWPE solutions in decahydronaphthalene at 135 °C. The MW was calculated using the Margolies equation [17]:

(10) $$Mw=5.37\cdot 10^{4}{\left[\eta \right]}^{1.49}$$

When preparing UHMWPE solution samples for subsequent measurements, the duration of dissolution is of great importance. With increasing polymer MW, the duration of the dissolution process increases. For complete dissolution of UHMWPE at 160 °C in 1,2,4-trichlorobenzene, 4.5–5.0 h are required.

In order to achieve complete dissolution of the samples, the studied UHMWPE grades were preliminarily dissolved in 1,2,4-trichlorobenzene at 160 °C for 5.0 h with constant stirring. To calculate the true MW characteristics of UHMWPE, the Mark–Houwink parameters for PS and PE, Equations (7) and (8), respectively, were used. In addition, the MW characteristics were also calculated relative to the PS standard using linear and cubic polynomial approximations.

**Figure 2:**
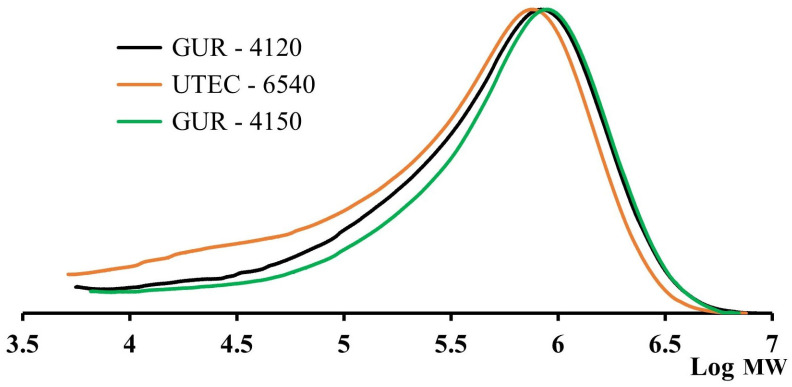
Results of measurements of the MWD of the studied UHMWPE samples, obtained by dissolution for 5 h.

The results of MWD measurements are presented in Figure 2, and the calculated molecular characteristics are collected in Table 1.

**Figure 3:**
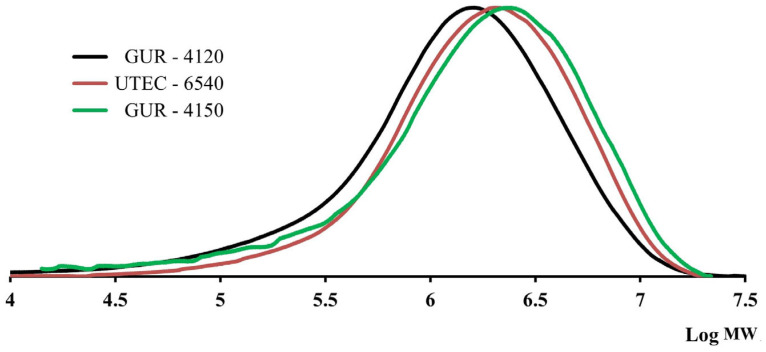
Results of measurements of the MWD of the studied UHMWPE samples, obtained by dissolution for 2.5 h.

The obtained data show that the MW characteristics of the studied grades do not correspond to the declared MW values of UHMWPE by the manufacturers. According to calibration No. 1, the MW of all grades is in the range of (6–8) · 10^5^ g/mol. The MWD is wide and is 6.4–9.6. In addition, the MWD curve of UTEC-6540-grade UHMWPE (Figure 2) has a significant low-molecular weight shoulder. Then, the studied UHMWPE samples were dissolved under the same conditions, but for 2.5 h. The MW characteristics of UHMWPE were calculated using the methods mentioned above. The obtained MWD curves for such preliminary sample preparation are shown in Figure 3. The MWD and MWD values obtained using this method of sample preparation are presented in Table 2.

As can be seen, the data in Table 1 and Table 2 differ significantly from each other. In addition, the results presented in Table 2 do not coincide with the MW values specified by the UHMWPE manufacturers, although in some cases they are closer to each other. Thus, the declared MW of GUR-4150-grade UHMWPE is 9.0 · 10^6^ g/mol, while the actual MW value of this grade is ~2.8 · 10^6^ g/mol (according to other calibrations 4.0 · 10^6^ or 9.4 · 10^6^). It can be assumed that, most likely, when determining the MWs of the UHMWPE grades, the Mark–Houwink equation correction factors for PE and PS, depending on both the nature of the polymers and solvent, and on the temperature, were not used. Instead, the MW of UHMWPE was determined relative to the PS standards without correction factors and using cubic approximation (calibration No. 3) instead of linear (calibration No. 2). This approach yields MW values more or less close to those declared by the manufacturer. 

Meanwhile, the use of cubic approximation in determining MW characteristics is incorrect. According to generally accepted concepts, the dependence of the intrinsic viscosity on MW with standard growth should be described by a linear function (in logarithmic coordinates).

It is also worth paying attention to the fact that all the studied samples have a wide MWD. Commercial UHMWPE as well as polyethylene and other polyolefins are usually polymerized using the heterogeneous Ziegler–Natta catalyst systems. These catalysts have several active sites with different activity. Therefore, this catalyst leads to broad MWDs of polymers synthesized.

In addition, polyethylene produced using single-site metallocene catalysts rarely has a very narrow molecular weight distribution. The MWD in this case can be in the range of 2.0–3.0.

The presence of molecular fractions with low molecular weight makes the MWD curve asymmetric. Therefore, because of the Ziegler–Natta catalyst with several active sites used by UHMWPE polymerization, the MWD curve is broad and asymmetric, and one can see a long tail in the area of low molecular weights.

The viscometry study confirms the correctness of the values obtained using GPC (calibration No. 1). The viscosity vs. MW of UTEC-6540-grade UHMWPE, obtained by GPC, is 2.6 · 10^6^ g/mol, which is close to the value of 2.2 · 10^6^ g/mol obtained on a viscometer.

**Figure 4:**
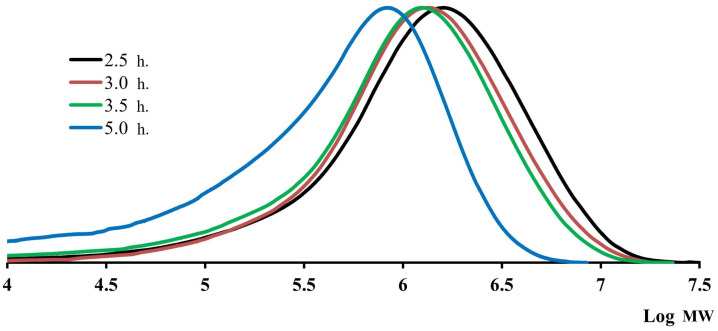
Shift of the MWD curves of the GUR-4120 sample depending on the duration during the preparation of the studied solutions.

As can be seen from the comparison of the data collected in Table 1 and Table 3, the MW characteristics differ significantly depending on the duration of dissolution, all other things being equal. Therefore, an additional study of the GUR-4120 sample was carried out. This sample was pre-dissolved for 2.5, 3.0, 3.5, and 5.0 h at 160 °C with constant stirring. The obtained MWD curves are presented for the studied samples in Figure 4.

The obtained MW and MWD characteristics, depending on the sample preparation conditions, are presented in Table 3.

As can be seen, the reduction in the dissolution time leads to a systematic increase in the “apparent” average MW and a narrowing of the MWD of the UHMWPE sample under study. Obviously, the same is typical for other UHMWPE grades.

Long-term dissolution leads to the appearance of a low-molecular weight “tail” of the polymer on the MWD curves. This is probably due to the partial destruction of UHMWPE. It can be assumed that the destruction is also facilitated by the presence of calcium or zinc stearates in industrial UHMWPE grades, introduced as plasticizing additives to improve the technological properties of the polymer.

As can be seen, there are serious methodological difficulties in measuring the MW of UHMWPE, caused by the poor solubility of PE even at high temperatures. Here, the overlap of two kinetic processes is significant—the rate of dissolution and the rate of destruction of PE. Each macromolecular chain is linked to other chains by a large number of entanglements. Even in a very dilute solution, there is a statistical probability that some entanglements are preserved longer than the characteristic time of chain disintegration. Therefore, it is impossible to be sure that two conditions are met simultaneously—complete dissolution and the invariance of the molecular composition of the sample during the entire experiment. Therefore, despite all possible precautions taken, the data presented in Table 1, as well as the manufacturer’s data, should be approached with caution. As will be shown below, this also applies to long-term measurements of the relaxation characteristics of UHMWPE.

### 3.2. Rheology of UHMWPE

#### 3.2.1. Creep Under Different Stresses

**Figure 5:**
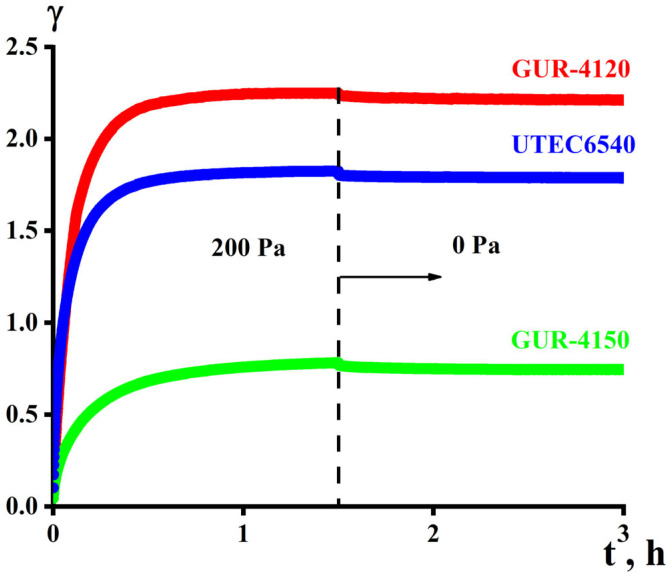
Creep (at σ = 200 Pa) of and elastic recovery for the three samples at 210 °C.

A typical example of the results of measuring creep and elastic recovery after removing the load is shown in Figure 5. The experiment consisted of loading the sample with a constant stress (in this example 200 Pa) for 1.5 h, after which the stress was ceased and the material was allowed to recover for 1.5 h.

It should be noted that longer experiments are impossible (and most likely unnecessary) because the UHMWPE samples begin to degrade rapidly, despite the use of the antioxidant.

Based on data such as those presented in Figure 5, it is possible to determine the equilibrium value of the ultimate strain γ* depending on the creep stress and the elastic modulus corresponding to the elastic recovery, which in all cases turns out to be insignificant. The existence of the ultimate strain γ* indicates not a model of viscoelastic behavior of the polymer due to weak elasticity, but a model of elastic–plastic behavior with a corresponding modulus of plasticity, P, expressed as follows:

(11) $$P=\sigma /\gamma^{*}$$

**Figure 6:**
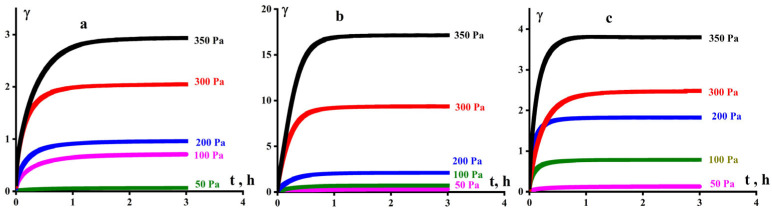
Creep of UHMWPE C for samples GUR 4150 (**a**), GUR-4120 (**b**), and UTEC 6540 (**c**), at 210 °C.

Experimental data demonstrating the creep of the studied samples (without elastic recovery) for various stresses are presented in Figure 6.

**Figure 7:**
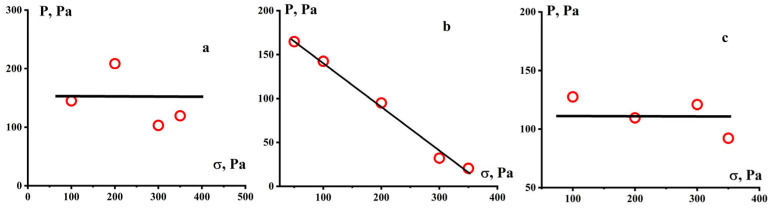
Moduli of plasticity for UHMWPE samples GUR 4150 (**a**), GUR-4120 (**b**), and UTEC 6540 (**c**).

The obtained values of the ultimate deformation correspond to the values of the plasticity modulus shown in Figure 7. Although the moduli of plasticity were obtained with a significant spread, it can be assumed that for the GUR-4150 sample, the modulus is 150 ± 50 Pa, for UTEC 6540–120 ± 30 Pa, but for the GUR-420 sample there is a clearly expressed strong dependence of the plasticity modulus on stress.

**Figure 8:**
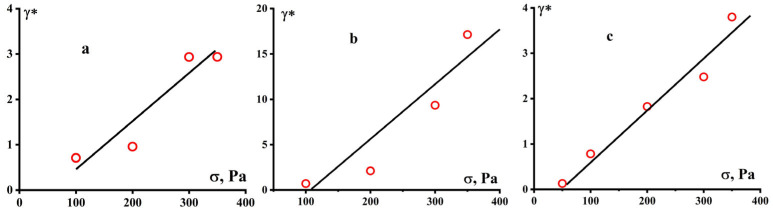
Dependence of the ultimate deformations for GUR 4150 (**a**), GUR-4120 (**b**), and UTEC 6540 (**c**), at 210 °C.

Meanwhile, one can see the difference in the levels of ultimate plastic deformations γ* of the GUR-4150 and UTEC samples not exceeding 4 units, and GUR-4120 reaching 20 units (Figure 8). This polymer also differs from the other two samples in that the plasticity modulus drops sharply to very low values with increasing stress, while for the two other PE samples the modulus is practically constant (Figure 7). Apparently, this is due to the contribution of the low-molecular “tail” to GUR-4120, detected during the MWD study for this polymer.

**Figure 9:**
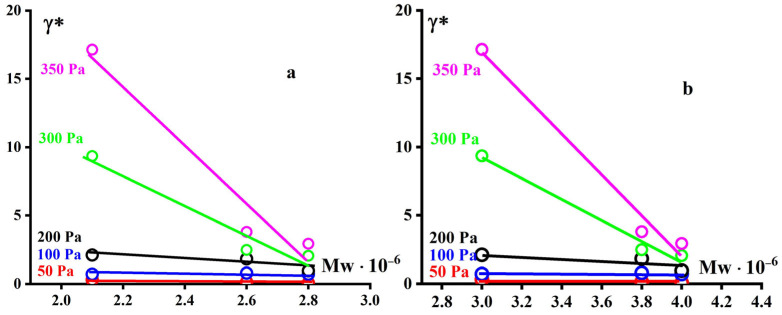
Dependence of the ultimate deformation on MW for GUR 4150, GUR-4120, and UTEC 6540 samples at 210 °C related to PE series (**a**) and related to PS-linear series (**b**).

The plasticity of the compared samples is an important technological factor, since the molding of UHMWPE items occurs during the plasticity mode, and not during the flow, which is typical for thermoplastics. The comparison of plasticity with MW is quite difficult due to the ambiguity of the definition of MW. However, standard measurements of the MWs (Table 2) indicate that GUR-4120 has a clearly lower MW compared to the other two samples. This corresponds to greater plasticity (Figure 9). Nevertheless, it seems that the main factor responsible for plasticity is the MWD of polymers, namely the presence of plasticizing low molecular fractions.

#### 3.2.2. Dynamic Modulus at Different Frequencies

In this section, the results of traditional measurements of frequency dependences of the components of the dynamic modulus of elasticity will be presented:

(12) $$G^{*}\left(\omega \right)=G^{\prime }\left(\omega \right)+iG^{\prime \prime }\left(\omega \right)$$

The storage modulus (or simply elastic modulus) G’(ω) and loss modulus G”(ω) in the region of linear viscoelastic behavior of the studied samples were determined at three temperatures of 170, 210, and 240 °C in the frequency range of 0.628–628 rad/s.

**Figure 10:**
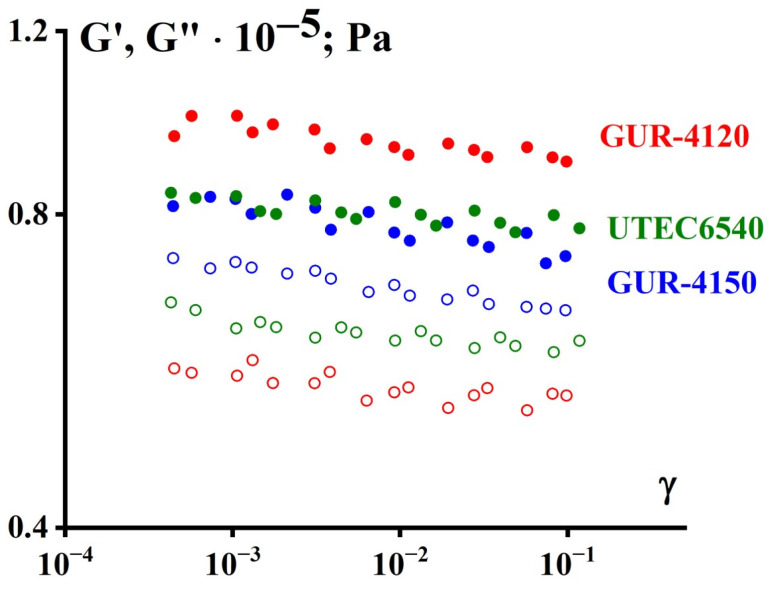
Elastic modulus components obtained at different deformation amplitudes (temperature 170 °C, frequency 6.28 rad/s); filled symbols—G’, open symbols—G”.

The amplitude dependencies of the components of the dynamic modulus were preliminarily measured at several frequencies to establish the limits of linearity. The obtained experimental results in the form of dependencies of the components of the dynamic modulus on the amplitude of deformation A under harmonic oscillations. They turned out to be the same for all the studied cases. Therefore, as an example, Figure 10 shows the amplitude dependencies of the components of the dynamic modulus for one series of measurements at a frequency of 6.28 rad/s and a temperature of 170 °C. At higher temperatures, linearity is maintained even up to higher deformation amplitudes.

The obtained results indicate that for at least up to an amplitude of the order of γ = 0.1, the linearity of the viscoelastic characteristics is maintained (the modulus components do not depend on the amplitude). Therefore, all measurements of G’(ω) and G”(ω) were subsequently carried out at a shear deformation amplitude equal to 0.1.

**Figure 11:**
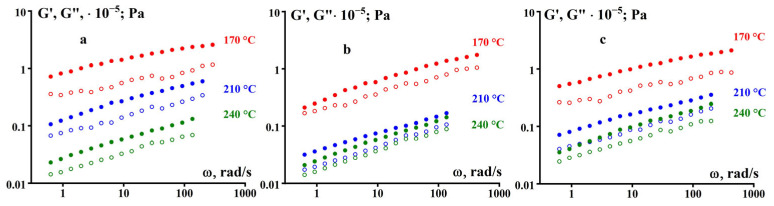
Frequency dependencies of G’(ω) (filled symbols) and G”(ω) (open symbols) for samples GUR-4120 (**a**), GUR-4150 (**b**), and UTEC 6540 (**c**).

The reproducibility of the results was confirmed by repeating the measurements three times, which showed a data spread of no more than 1%. The results of measuring the frequency dependencies of the dynamic modulus components are presented in Figure 11.

**Figure 12:**
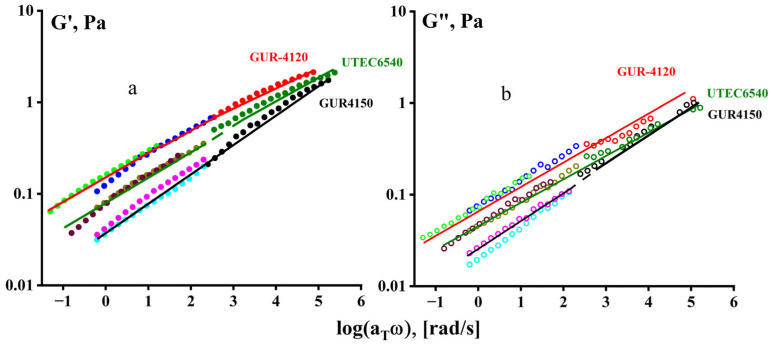
Temperature–frequency superposition of the elastic modulus (**a**) and loss modulus (**b**) for samples GUR-4120, GUR-4150, and UTEC 6540. Master curves are built for 240 °C.

The temperature–frequency reduction in the obtained data is shown in Figure 12, where the reduction factor a_T_ was found empirically by combining the curves (from Figure 11) by shifting along the frequency axis. As can be seen, when moving from 170 °C to 240 °C, there is a jump in the temperature dependence, indicating a change in the relaxation properties of the polymer in this region.

From the data on the evaluation of the frequency dependencies of the elastic modulus, the following conclusions can be made about the relaxation state of the studied polymers. Firstly, at any temperature and frequency, G’ > G”, and in no case is a transition or even approach to the terminal region of the viscoelastic state observed. The slope of almost all dependencies is 0.33 ± 0.02, typical for weakly structured systems such as gelling systems [18]. This corresponds to the transient region in the sol–gel transition above the gel point, i.e., there is a quasi-stable structure. Thus, we can confidently conclude that there is no steady-state viscous flow. Secondly, in the low-frequency region, we can presumably think about the plasticity. The high-frequency region also is rather far from the rubbery relaxation state.

Long-time behavior (corresponding to the low frequency region) was described in the previous section. However, it would be interesting to compare characteristic relaxation times of a linear low-molecular weight PE with the expected terminal zone of UHMWPE.

For this purpose, the relaxation properties of a low-molecular PE grade in the terminal region of viscous flow at 210 °C were measured as a benchmark. The purpose of this experiment is to obtain values of the characteristic relaxation time θlow in the region, in which Maxwellian dependencies are fulfilled:

(13) $$G^{\prime }\left(\omega \right)=G_{0}\frac{{\left(\omega \theta \right)}^{2}}{1+{\left(\omega \theta \right)}^{2}}$$

(14) $$G^{\prime \prime }\left(\omega \right)=G_{0}\frac{\left(\omega \theta \right)}{1+{\left(\omega \theta \right)}^{2}}$$

Then, θ_low_ for the terminal (flow) relaxation state can be calculated as follows:

(15) $$\theta_{low}=\frac{G^{\prime }}{G^{\prime \prime }\omega }$$

This formula is equivalent to the following relation:

(16) $$\theta_{low}=\frac{\varsigma }{\eta }$$

which follows from two following expressions:

(17) $$G^{\prime }=\varsigma_{0}\omega^{2}$$

(18) $$G^{\prime \prime }=\eta_{0}\omega$$

where ζ_0_ and η_0_ are coefficients in the $G^{\prime }\left(\omega \right)$ and $G^{\prime \prime }\left(\omega \right)$ dependencies, respectively.

**Figure 13:**
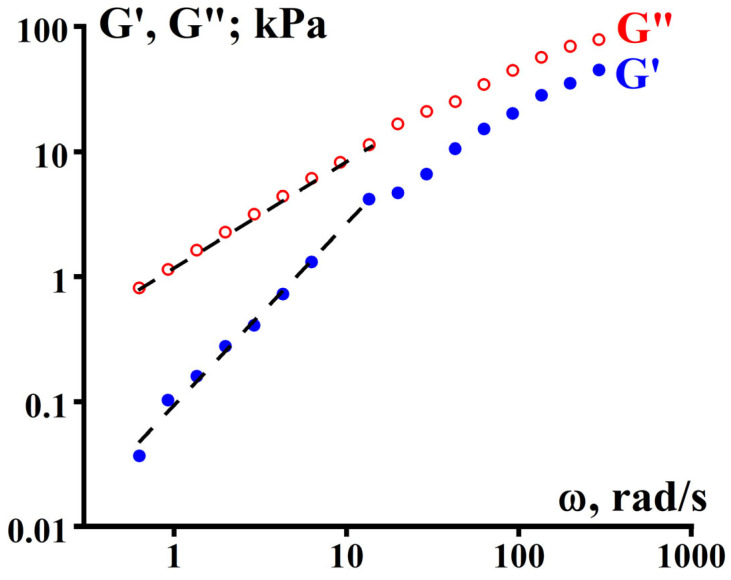
Visco-elastic properties of the linear low-MW PE at 210 °C.

The corresponding experimental data are presented in Figure 13.

In the case under consideration, ζ_0_ ≅ 100 Pa · s^2^ and η_0_ ≅ 10^3^ Pa · s. Consequently, θ_low_ ≅ 0.1 s.

The lower (terminal) limit of the relaxation time values of UHMWPE, M_high_ with M = 3.0 · 10^6^, can be estimated using the simplest relationship for the dependence of viscosity on MC, assuming that the elastic modulus does not depend (or weakly depends) on MW.

(19) $$\theta_{high}=\theta_{lkow}{\left(\frac{M_{high}}{M_{low}}\right)}^{3.5}$$

Then, for M_low_ of the order of 3.0 · 10^4^, we obtain that θ_high_ is of the order of 6.2 · 10^7^ s (>17 h). Thus, relaxation times of the UHMWPE always lie far above the terminal zone (as seen in Figure 7) This value significantly exceeds the thermal stability time of UHMWPE, and this shows that no UHMWPE samples can be in the flow region and are only capable of plastic deformations. This is exactly what was observed in the creep experiments described above.

It may be rather interesting to try estimating what viscosity corresponds to this relaxation time. This estimate can only be quite rough. However, let us we estimate the elastic modulus, G_e_ from the elastic deformation, which is no more than 0.1 (from Figure 5). Then, the elastic modulus should be of the order of 3.5 · 10^4^ Pa. For relaxation time θhigh ∼6.2 · 10^7^ s, a rough estimate of the viscosity, η = G_e_θ_high_ gives a value of the order of ∼10^11^ Pa · s. Such a value corresponds to the glassy state, and viscous flow with such a viscosity value can hardly be actually observed.

## 4. Conclusions

Direct measurements of the molecular characteristics of several samples of UHMWPE by the GPC method showed that the results strongly depend on the method of calibration in the GPC measurements. In addition, the obtained average MW values do not agree with the characteristics provided by the manufacturer.

Although UHMWPEs belong to the class of thermoplastic polymers, they cannot flow. It was found that the observed irreversible deformations must be considered as plastic deformations. This means that under the action of applied shear stresses, irreversible shear reaches a certain level depending on the stress but does not increase with time in contrast to flow, defined as an unlimited increase in deformation. Plastic deformations for high-molecular weight UHMWPE samples reach several units, although for the sample with the lowest MW, the ultimate plastic deformations can reach 20 units. This newly discovered type of rheological behavior of long-chain polymers is associated with very long relaxation times of the terminal zone (corresponding to flow), which greatly exceed the thermal stability time of the polymer. This also explains the unattainable steady flow regime for UHMWPE, since attempts to increase the duration of shearing at high temperatures lead to thermal destruction of the polymer.

Thus, the processing of UHMWPE for obtaining any products can be carried out only in the mode of plastic deformations.

Quantitative comparison of the obtained rheological characteristics with the MWs of the UHMWPEs is difficult (or not entirely reliable), since direct measurements of the molecular characteristics by the GPC method strongly depend on the method of calibration in the GPC measurements, and the obtained MW values cannot agree with the characteristics provided by the manufacturer.

## Figures and Tables

**Table 1:** Molecular weight characteristics of the studied UHMWPE grades calculated using three calibration methods, with preliminary dissolution for 5 h.

Sample	Data of Manufacturers M_w_∙10^−6^	Calibrated by PE		Calibrated by PS—Linear		Calibrated by PS—Cubic	
		M_w_∙10^−6^	M_w_/M_n_	M_w_∙10^−6^	M_w_/M_n_	M_w_∙10^−6^	M_w_/M_n_
GUR-4120	4.0	0.8	6.5	1.0	7.7	1.0	6.9
UTEC6540	9.0	0.6	8.0	0.8	9.6	0.7	7.7
GUR-4150	8.0	0.8	6.4	1.1	6.8	1.0	7.0

**Table 2:** Molecular weight characteristics of the studied UHMWPE grades calculated using three calibration methods, with preliminary dissolution for 2.5 h.

Sample	Data of Manufacturers M_w_∙10^−6^	Calibrated by PE		Calibrated by PS–Linear		Calibrated by PS–Cubic	
		M_w_∙10^−6^	M_w_/M_n_	M_w_∙10^−6^	M_w_/M_n_	M_w_∙10^−6^	M_w_/M_n_
GUR-4120	4.0	2.1	4.9	3.0	5.8	5.9	12.1
UTEC6540	9.0	2.6	2.9	3.8	3.3	7.7	7.9
GUR-4150	8.0	2.8	5.2	4.0	6.1	9.4	15.2

**Table 3:** Dependence of MW characteristics of GUR-4120-grade UHMWPE on dissolution duration.

Dissolution Duration, h	Mw∙10^−6^	Mw/Mn
2.5	2.1	4.9
3.0	1.8	4.0
3.5	1.6	5.6
5.0	0.8	6.5

## Data Availability

The data are contained within the article.
